# Microbiota Transplant in the Treatment of Obesity and Diabetes: Current and Future Perspectives

**DOI:** 10.3389/fmicb.2020.590370

**Published:** 2020-11-12

**Authors:** Michael Napolitano, Mihai Covasa

**Affiliations:** ^1^Department of Basic Medical Sciences, College of Osteopathic Medicine, Western University of Health Sciences, Pomona, CA, United States; ^2^Department of Health and Human Development, Stefan Cel Mare University of Suceava, Suceava, Romania

**Keywords:** gut microbiota, obesity, metabolic syndrome, T2DM, SCFA

## Abstract

A wealth of evidence has revealed the critical role of the gut microbiota in health and disease. Many chronic diseases have been associated with gut microbiota imbalance in its composition, diversity and functional capacity. Several types of interventions have been shown to correct microbiota imbalance and restore the beneficial metabolic outcomes of a normal microbiota. Among them, fecal microbiota transplantation (FMT) is an emergent, promising technology employed to improve clinical outcomes of various pathological conditions through modifications in the gut microbiota composition. FMT has been used successfully as a treatment option in recurrent *Clostridium difficile infection*, a condition characterized by severe gut microbiota dysbiosis. However, the potential usage of FMT in other microbiota-associated conditions different from *C. difficile* such as metabolic syndrome or obesity that are also marked by gut dysbiosis is still under investigation. Furthermore, the contribution of the gut microbiota as a cause or consequence in metabolic disease is still largely debated. This review provides critical information on the methodological approaches of FMT and its technological innovation in clinical applications. This review sheds light on the current findings and gaps in our understanding of how FMT can be used as a future biotherapeutic to restore microbial homeostasis in amelioration of obesity and diabetes.

## Introduction

Outnumbering the human genome by 150:1, the bacterial microbiome has played a crucial role in the development and evolution of human beings throughout time (Ejtahed et al., 2016). The gut microbiome is rich with many bacterial species as well as with a variety of fungi, yeasts, and viruses. It hasn’t been until recently that modern technology has allowed scientists to really begin to understand their purpose and overall importance within the human body. One of the most important discovery in microbiome research is the concept that bacterial diversity impacts human health and disease (Khanna and Tosh, 2014). From birth to death, the turnover of the microbiome is relatively constant (Khanna and Tosh, 2014). However, the diversity and adaptability of the microbiome and its by-products change drastically throughout one’s lifetime (Martinez et al., 2013). For example, the richness and diversity of the microbiome has been shown to peak during mid-adolescence and decline with aging. The aforementioned uniformity in bacterial species ultimately increases one’s susceptibility to many pathological processes. Thus, the inevitable decline in bacterial diversity in senescence might be responsible for certain diseases associated with advanced age (Khanna and Tosh, 2014). Notwithstanding the great diversity of different bacterial strain richness and ratio exhibited amongst individuals, fecal samples indicate that there is significant adaptability in the actual composition of the microbiome across one’s lifetime (Ejtahed et al., 2016). This does not mean that variability and alterations of an individual current microbiota composition cannot and does not occur. Similarly, there is great diversity in the microbiome composition between individuals due to a multitude of factors. For example, vaginal vs. cesarean birth, breastfeeding, diet, environmental conditions and antibiotic use are among the main factors that can greatly impact the composition of the microbiome, ultimately leading to bacterial disequilibrium and variations in prognostic outcomes (Ejtahed et al., 2016). Indeed, microbial imbalance has been associated with a broad spectrum of pathologies ranging from obesity, metabolic syndrome and inflammatory bowel disease (IBD) to autoimmunity and mental health disorders (Lee et al., 2018). Disruption in the gut microbial ecosystem has been documented to have many potential causes including antibiotic overuse, increase consumption of high fat diet and high levels of systemic inflammation due to over exposure of lipopolysaccharide (LPS) by gram negative bacteria (Vallianou et al., 2018). A host of other environmental factors have been identified as potential triggers for an altered gut microbiome such as organic pollutants, foodborne toxins and preservatives, and sanitation-related chemicals commonly used in agriculture. All of these have been shown to significantly impact the composition of the gut microbiome, causing significant changes in the microbial metabolic activity and increasing the risk of gastrointestinal (GI)-related diseases (Sonnenburg and Sonnenburg, 2019). The exact mechanisms linking gut microbiome imbalance to metabolic syndrome and other pathologies are not completely understood. Current studies focusing on this relationship have provided promising avenues of research that include altering the efficiency of digestion, exploring the role of bacteria in producing short chain fatty acids (SCFAs) along with other metabolites and studying the impact of the diet and microbiome transplants on altering or restoring the microbial composition profile. The remodeling of the gut microbiome composition via diet, genetics and other medical procedures such as fecal microbiota transplantation (FMT) could lead to new strategies of treatment for many common disease processes (Marotz and Zarrinpar, 2016). Therefore, in this review, we will focus on the methodology and principles of microbial reconfiguration via FMT. In this process, we will briefly address the role of gut microbiome in obesity and metabolic syndrome followed by the role of FMT in the therapeutic outcomes of these conditions. Finally, we will discuss the current gaps, challenges and limitations in the use of FMT as a treatment modality, as well as highlight potential next steps for its utilization in the future treatment of metabolic diseases.

### The Commensal Bacteria

With over 1000 known species to date and many more likely to be discovered in the coming years, the GI tract harbors the greatest abundance and diversity of bacterial species in the human body (Villanueva-Millan et al., 2015). Though it constantly adapts and evolves concurrently with lifestyle changes, it hasn’t been until recently that modern technology has allowed us to uncover the potential therapeutic implications offered by the gut microbiome. PCR-denaturing gel electrophoresis, microarrays, fluorescence *in situ* hybridization and metagenomics, are only a handful techniques used to identify, map and study the microbiome (Vallianou et al., 2018). In particular, the discovery of new bacterial strains can be attributed to the rise in new genomic technology such as 16S rRNA gene sequencing that allows detection of bacterial strains that exhibit specific functionality within the human gut, with possible major implications in metabolism and homeostasis (Clavel et al., 2014). It is important to note that the species identified via this method are confirmed by cross referencing them with a database of bacterial species that have already been discovered, thus limiting the identifiable species to those that have been previously sequenced (Ejtahed et al., 2016). This leaves a large group of unrecognized species that are still unaccounted for (Ejtahed et al., 2016).

The commensal bacteria are strains found within the human body that flourish symbiotically in the gut as well as other organ systems. The variety of these different species can change based on several factors such as temperature or pH of the environment (Swidsinski et al., 2005; Gu et al., 2013; Donaldson et al., 2016; Scheithauer et al., 2016). Of the many different commensal strains in the human body, the vast majority include the *Firmicutes* (Gram negative), *Bacteroidetes* (Gram positive), *Verrucomicrobia* (Gram negative), *Actinobacteria* (Gram positive), and *Proteobacteria* (Gram negative) species (Swidsinski et al., 2005; Gu et al., 2013; Donaldson et al., 2016; Scheithauer et al., 2016). The proximal GI tract hosts mainly *Proteobacteria* and *Firmicutes* whereas *Bacteroidetes*, *Verrucomicrobia*, *Actinobacteria*, and *Proteobacteria* are housed by the more anaerobic distant GI and colon (Swidsinski et al., 2005; Gu et al., 2013; Donaldson et al., 2016; Scheithauer et al., 2016). Of these top five species, the most abundant is the *Firmicutes*, which exceeds 200 genera including *Mycoplasma*, *Bacillus*, and *Clostridium*, encompassing nearly 65% of the entire flora (Ejtahed et al., 2016). These top species account for roughly 97% of the entire microbiome in most individuals (Benson et al., 2010; Human Microbiome Project Consortium, 2012), though there is variation in their respective quantitative representation (Villanueva-Millan et al., 2015). In addition to their abundance in the human GI tract, commensal strains also reside on the skin, genitourinary tract, respiratory tract and other mucosal surfaces (Villanueva-Millan et al., 2015). Diet plays a major role in the individual diversification of commensal strains in the human body. For example, *Bacteroides* has been associated with a low intake of carbohydrates, yet high in dietary fat and animal protein (Wu et al., 2011; Gorvitovskaia et al., 2016). The relationship between bacterial specificity and diet has drawn significant attention in the study of metabolic diseases (Wu et al., 2011; Gorvitovskaia et al., 2016). Despite the rise in scientific interest, most of the human studies conducted thus far have described a relationship represented primarily by correlation rather than causation when it comes to specific bacterial strains and the occurrence of metabolic disease (Wu et al., 2011; Gorvitovskaia et al., 2016).

### Host-Commensal Symbiosis

Commensal microbiota live in a symbiotic relationship with their host. Both the bacteria and the host organism support one another through various means that cannot be otherwise achieved individually. As such, commensals have the task of helping the host maintain homeostasis through metabolic processes like carbohydrate fermentation and SCFA production (Leustean et al., 2018). In exchange, the host offers a primarily anaerobic environment (i.e., the GI tract) and a wide range of nutrients, like complex fibrous carbohydrates, that are necessary for bacterial survival. Many of the nutrients that the bacteria require are diet derived (Woting and Blaut, 2016). Some of these nutrients include indigestible fibers such as cellulose, inulin, xylin, and pectin, which originate from plant matter (Han and Lin, 2014) and for which humans lack the required digestive enzymes necessary for their catabolism and metabolization, thus relying almost exclusively on these bacteria. Therefore, this mutualism between humans and microorganisms in the gut is of major importance to both the host organism and the bacteria (Han and Lin, 2014). The bacteria also grant significant advantages to the host by performing many other bacteria-specific metabolic reactions such as carbohydrate fermentation as well as the synthesis of vitamin K, reactions that the host is incapable of performing alone (Woting and Blaut, 2016). Along with indigestible plant fibers, local mucins and desquamated epithelial cells are utilized by local bacteria for sustenance and removal from the GI tract (Woting and Blaut, 2016). Similar to its role in digestion of indigestible nutrients, which produce short-chain fatty acid metabolites as a byproduct and vitamin synthesis, commensal gut bacteria are involved in bile acid dihydroxylation and mucosal immune system maintenance (Aron-Wisnewsky et al., 2019).

The bacterial fermentation of indigestible carbohydrates yields specific molecular byproducts such as SCFA (Yoshida et al., 2018) that are used as energy source by the host organism for daily metabolic requirements. By fermenting fibrous carbohydrates, resident bacteria allow the host to fully utilize the otherwise inaccessible nutritional value of these different plant fibers and generating as much as 10% of the overall caloric intake, or 200 kcal/day in some individuals (Okubo et al., 2018). Notably, some studies have shown that obese individuals display an increase in SCFA production in both fecal and plasma compared to lean individuals, which suggests a possible connection between SCFA production and obesity (Okubo et al., 2018). In addition, given their role in providing energy, SCFA are important players in maintaining gut barrier integrity, in maintaining glucose homeostasis, immune system regulation, and parasympathetic nervous system (PNS) activation within the GI tract and transcription regulation of host genes (van Olden et al., 2015). For example, butyrate and propionate, two main SCFA, have been shown to assist in the differentiation of regulatory T cells (Tregs) as well as with other immune cell differentiation in the GI tract. Acetate, the most abundant SCFA that binds to G-protein coupled receptors FFAR2 and FFAR3 expressed in various tissue such as muscle, adipose, pancreas, liver and intestine, has been implicated in the activation of the PNS (Yoshida et al., 2018). The latter effect is of great interest due to the possible role of acetate in obesity and hyperphagia (Yoshida et al., 2018). In the presence of glucose within the gut, the PNS is responsible for the secretion of insulin in order to maintain blood glucose levels. Lastly, SCFA have also been documented for their role in epigenetic modulation within the GI tract (Okubo et al., 2018). More specifically, SCFA upregulate the transcription of certain genes within the host’s genome by means of signal transduction altering pathways (Okubo et al., 2018). As previously mentioned, B vitamin complex and vitamin K are also produced by the gut bacteria (Leustean et al., 2018). These vitamins play a role in degrading biliary acids and steroids which could have some clinical significance in treating patients with certain hyperlipidemia conditions (Leustean et al., 2018).

An additional resource of importance to commensal bacteria is the mucus produced by the goblet cells dispersed throughout the epithelial cells of the GI tract. The bacteria selectively use the glycans derived from the mucus as a growth substrate (Sonnenburg et al., 2010). This is accomplished by a complex two-component system of proteins that are involved in gene expression, specifically those that involve glycan metabolism (Lynch and Sonnenburg, 2012). The two-component system of glycan metabolism involves polysaccharide receptor proteins on the cell membrane that, when bound by a metabolite, which in this case is a polysaccharide (glycan), elicits a phosphorelay signal in a multistep relay process via cytoplasmic transducer proteins to the cell genome (Lynch and Sonnenburg, 2012). Once the relay signal reaches the cell genome, transcription factors then upregulate cell transcription leading to protein expression and cell growth (Lynch and Sonnenburg, 2012). The glycan metabolites act as the ligand to these polysaccharide receptor proteins on the cell membrane which when metabolized trigger the signal cascade that leads to cellular growth. Proteins, amino acids and peptides are also utilized by the gut bacteria in the distal colon, but to a lesser degree (Woting and Blaut, 2016). Additionally, the gut microbiome is partially responsible for immune system homeostasis within the mucosal membrane of the gut. As such, the microbiome helps facilitate the secretion of IgA immunoglobins in the mucosal membrane lining the GI tract, a first-line defense barrier against many foreign pathogens (Khanna and Tosh, 2014). When exogenous pathogens enter the gut lumen, immune cells located in the gut mucosa secrete IgA which coat the invading pathogen/substance. The IgA bound-pathogen is a trigger for circulating immune cells to perform cell-mediated destruction of the molecule and ultimately prevent pathogenic infection. Also gut bacteria produce antimicrobial peptides, such as RegIII and alpha-defensins that protects host mucosa and has a role in chemostasis and toll-like receptors (TLRs) signaling. This immunological functionality expressed by the gut bacteria is pivotal to maintaining the integrity of the mucosal membrane and health of the host organism, without which the GI disease as a result of pathogenic infection could persist (Khanna and Tosh, 2014). If foreign pathogens are able to interact directly with the mucosal membrane without immune cell interception beforehand they can’t only directly invade the protective cell membrane, infecting the host but the repetitive damage to the primary barrier between the organism and the outside world can cause the host to become more vulnerable to future infection. The mucosal immune cells prevent this by actively identifying and destroying these pathogens before mucosal invasion can ensue.

## Fecal Matter Transplant (FMT)

Various methods have been employed to manipulate the gut microbiome as a way to explore their functionality and to develop new therapeutic modalities for clinical applications using them. The addition of prebiotics (which induce bacterial growth) and probiotics (non-pathogenic beneficial strains of various microorganisms) are among the most popular and widely used modulators of gut microbiome composition (Marotz and Zarrinpar, 2016). Although the results of studies in humans show some health benefits from the use of prebiotic and probiotic supplements, there is still a great deal of speculation as well as inconsistencies among findings. In a systematic review by Tenorio-Jimenez et al. (2020), it was concluded that although there has been some beneficial effects from the use of probiotics in various studies, these effects are marginal in comparison to that of dietary improvement and medical pharmaceuticals when used to treat GI disease. Testing different bacterial strains in various quantities is required in order to prove if the addition of specific prebiotic or probiotic compounds results in a significant improvement of parameters associated with certain pathologies or if any comparison can be made to current drug-based treatment modalities (Preidis and Versalovic, 2009). Another non-antimicrobial, albeit more controversial approach, that has garnered recent attention is the FMT procedure. The FMT has the propensity to not only improve the functioning of the commensal host bacteria, but also to completely remodel the entire host microbiome. FMT achieves this by altering the actual composition and ratio of the resident commensal species present in the host. The metabolic capabilities of the altered microbiome can differ greatly from the previously inhabited strains that can result in improved physiological functionality (van Nood et al., 2013).

Fecal microbiota transplantation is not a new procedure. Fecal transplantation has been used since the 4th century A.D, primarily by the Chinese, who gave suspensions of fecal matter to patients suffering from chronic diarrhea (Zhang et al., 2012). Similarly, FMT was used in the 16th century and in the 1950’s to treat similar GI maladies (van Nood et al., 2013). Despite these recorded practices, there is an obvious social stigma surrounding its overall use. Generally, the idea of FMT itself is unappealing. However, recent surveys from interviewing *Clostridium difficile*-infected individuals showed, that despite FMT being considered an unorthodox and uncommon procedure, patients were still willing to try it nonetheless (Jayasinghe et al., 2016). Currently, the FDA has only approved the use of FMT as a treatment modality for severe, recurrent *C. difficile* infections. However, research is currently being conducted to determine the efficacy of its potential use in the restoration of gut bacteria disequilibrium that is associated with obesity, metabolic syndrome and IBD (Zhang et al., 2012).

In terms of risk, FMT is considered a very safe procedure; however, some adverse effects have been documented. Minor side effects range from diarrhea and constipation to fever and abdominal pain, all of which last no longer than a week post-treatment (Grehan et al., 2010). Any reported deaths succeeding FMT are few-and-far-between and is often a result of comorbidities with unrelated disease processes, and are not related to the actual FMT procedure itself (Grehan et al., 2010). Determining the etiology of malignant reactions post-FMT is quite difficult due to the complexity in understanding whether the observed reactions are the result of an underlying disease process present before the treatment or if they are a response to the fecal transplant itself (Marotz and Zarrinpar, 2016). Theoretically, the transfer of infectious pathogens from donor to recipient can and does occur, but the intense pre-treatment screening process greatly minimizes this risk. The at-risk population for fatal adverse reactions are the immunocompromised, such as HIV patients and patients under immunosuppressive drug therapy. This is primarily due to their debilitated immune system, as a weakened immune system increases the likelihood of pathogenic transfer and subsequent infection from the donor sample (Dailey et al., 2019). There have been two reported cases of FMT patients who contracted extended-spectrum beta-lactamase (ESBL)-producing *Escherichia coli* bacteremia, one of which succumbed to the infection (DeFilipp et al., 2019). The patients received the same donor sample that genome sequencing later identified as the reservoir for this ESBL *E. coli* infection (Gutin et al., 2019). Although the donor was properly screened before the procedure, and lacked high risk factors, the recipients still developed bacteremia, which reveals that there is a potential infection risk with FMT regardless of the pre-treatment screening regimen (Gutin et al., 2019). Despite being a generally safe procedure, and armed with rigorous pre-treatment screening protocols, FMT still has the potential for unforeseen complications as can be expected with any medical procedure. A slightly more controversial risk associated with FMT is the development of obesity or chronic autoimmune disease after treatment (Grehan et al., 2010). Essentially, if an obese donor transfers the microbiome to a lean individual, there is the possibility that the lean individual may become obese. This theorized outcome still warrants further study due to the lack of significant literature support. Likewise, there has been research supporting a relationship between the gut microbiome and obesity/metabolic disease which has gained significant attention in the scientific community.

### FMT Procedure

Before an FMT procedure is performed, a viable fecal donation must first be obtained from a heavily screened individual. Typically, fecal donors are either close family members or friends with a known medical history. However, it is not uncommon to receive a donation from an unrelated donor (El-Matary et al., 2012). Before a fecal donation is obtained, the donor must undergo a battery of medical testing that includes hematological tests and fecal sample analysis in order to check for disease causing pathogens, malignancies, autoimmune disease, and other familial diseases (Preidis and Versalovic, 2009). The pre-treatment medical screening is critical because a medically compromised donor has the potential to transfer disease to the transplant recipient (Weissman and Coyle, 2012). Despite the extensive screening and questioning process of donors, the risk of transferring a potentially deadly disease is still high (Preidis and Versalovic, 2009). Similarly, the likelihood of developing a post-treatment infection, unrelated to the health status of the donor, is also a danger. Currently, blood screening protocols typically include a complete blood count, liver function test, HIV, CMV, EBV, syphilis, and hepatitis A, B, and C detection tests while the stool screening components encompasses PCR and immunological methods based tests for *C. difficile* toxin PCR, *Cyclospora*, *Isospora*, *Cryptosporidium*, *Giardia* antigen, HTLV I/II, and *Helicobacter pylori* (Weissman and Coyle, 2012). However, if the donor is sexually intimate with the recipient, certain sexually transmitted infection tests could be omitted (Weissman and Coyle, 2012).

Once the donor has passed the preliminary screening and questioning, the donor transplant sample can be obtained. One commonly used protocol for sample extraction is the Amsterdam Protocol, which uses a ratio of 200–300 g of stool dissolved in 500 g of a sterile saline solution within 6 h of expulsion (Preidis and Versalovic, 2009). In addition to sterile saline, other mediums such as water, milk, and saline combined with psyllium have been used as fecal diluents, all producing similar results (Gough et al., 2011). Upon extraction, the sample is frozen until its transplantation into the recipient (Preidis and Versalovic, 2009). There has been some debate on whether the use of fresh versus frozen samples impacts the overall efficacy of the treatment with current literature showing that both fresh and frozen samples have yield similar results (Smits et al., 2013). However, frozen samples remain the preferred method due to their practicality (Hamilton et al., 2012).

The fecal transplantation procedure varies to a great degree across different institutions and users (Marotz and Zarrinpar, 2016). Despite several techniques being practiced, the protocol is relatively easy to perform from a methodological perspective. The process begins with a reduction in the transplant recipient’s commensal flora, typically achieved with a multi-dose antibiotic therapy of vancomycin or doxycycline (Grehan et al., 2010). The purpose of this heavy pre-procedural antibiotic treatment is to eliminate the current strains harbored in the recipient so that the newly transplanted bacteria can thrive without competition for space and resources (Smits et al., 2013). Along with the antibacterial treatment, the host is often given a polyethylene glycol colon wash which has been shown to enhance the colonizability of the newly transplanted bacteria (Marotz and Zarrinpar, 2016). Once the recipient’s gut is cleansed of the thousands of previously inhabited bacterial colonies, the donor microbiome is ready to be transplanted. There are several common routes of fecal donor administration used in practice. The current gold standard is the colonoscopic and duodenal fecal infusion method both of which have been shown to yield the greatest relief of symptoms in *C. difficile* infected individuals (El-Matary et al., 2012). Other tried routes such as esophagogastroduodenoscopy, nasogastric tube and nasojejunal have been less successful in providing symptom relief (Weissman and Coyle, 2012).

**FIGURE 1:**
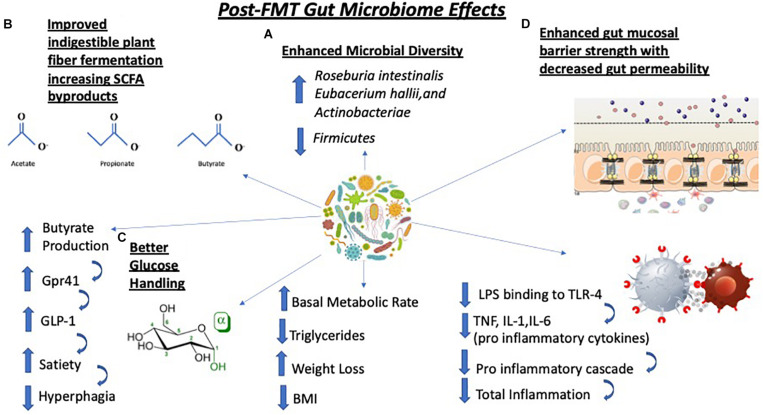
Fecal microbiota transplantation elicits a myriad of physiological changes such as **(A)** an increase in microbial diversity with specific increases in *Roseburia intestinalis*, *Eubacterium hallii*, and *Actinobacteria* as well as decreases in levels of *Firmicutes* species; **(B)** improvements in the fermentation of indigestible polysaccharides that generate short chain fatty acid (SCFA) such as butyrate. Increases in butyrate production trigger the G-protein receptor 41 (Gpr41) signaling cascade which causes secretion of glucagon-like peptide-1 (GLP-1), a key peptide regulating satiety, thus leading to decrease in food consumption; **(C)** better glucose handling which improves host metabolism, lowers triglycerides, triggers weight loss and lowers body mass index (BMI); **(D)** strengthening of a previously weakened gut epithelial barrier. This is due to less lipopolysaccharide (LPS), an endotoxin, binding to TLR4 on immune cells. which lowers the levels of pro-inflammatory cytokines TNF, IL-1 and IL-6 resulting in decreased inflammation. TNF-α, tumor necrosis factor; IL-1, interleukin-1; IL-6, interleukin-6.

In a study by Ramai et al. (2020) comparing various routes of administration of fecal transplantation between colonoscopic, nasogastric, capsule and enema in the treatment of recurrent *C. difficile* infection, there was a greater cure rate with colonoscopic (94.8%) administration and capsular (92.1%) compared to nasogastric (78.1%) and enema (87.2%). Although capsular administration was comparable in cure rate to colonoscopic administration, the use of capsules in this study had a smaller sample size when compared to colonoscopic (Ramai et al., 2020). Another example of the use of colonoscopic fecal transplantation is seen in a study measuring the efficacy of FMT in the treatment of Crohn’s disease in which donor recipients received a single-dose fecal transplant. Although the cohort was small (*n* = 10), three of the patients reported modest relief in clinical symptoms and exhibited altered gut microflora consistent with the strains common in the donor’s microbiome (Gutin et al., 2019). Figure 1 depicts the FMT process from both the donor’s perspective as well as the recipient’s, highlighting the key steps in both pre-treatment protocols.

## FMT and Obesity

The world is currently facing an unprecedented increase in the number of overweight and obese individuals (Chang et al., 2019). A staggering 33% of the world’s population over the age of 20 is overweight [body mass index (BMI) 25–30 kg/m2] or obese (BMI > 30 kg/m2). According to the World Health Organization these numbers are projected to grow in the coming years (Zhang et al., 2012).

The factors implicated in the development of obesity are complex and multifocal. Several studies point to genetic variability amongst obese individuals, specifically in the fat mass and obesity-associated (FTO) gene region of chromosome 16 coding for the FTO protein alpha-ketoglutarate-dependent dioxygenase (Dailey et al., 2019). This enzyme is directly involved in the regulation of hunger in the hypothalamus leading to increase food consumption and a higher propensity for obesity (Dailey et al., 2019). However, there is a strong consensus that the current epidemic levels of overweight and obesity are due to a complex interaction of genetics and environment which includes poor dietary habits, increased availability of dense calorie foods and lack of physical activity (Huang and Hu, 2015). The detrimental effects of obesity to human health is well documented. Numerous studies link obesity to a host of pathologies such as diabetes, hypertension, and metabolic syndrome, to name a few (Zhang et al., 2012). However, despite obesity’s ravaging effects on health, weight loss is one of the most difficult health pursuits one can attempt. It takes a great deal of patient compliance and dedication to overcome negative lifestyle habits such as overeating, poor nutrition, and lack of exercise. Even if an individual successfully manages to lose weight, there is often a weight-gain rebound effect triggered by the increased post-diet hunger leading to weight regain due to metabolic adaptation, thus perpetuating the cycle further. Additionally, if the individual has a high genetic predisposition toward an obesity, diet and exercise might not reverse the metabolic derangements already done. In light of this, continuous efforts have been dedicated toward finding ways to slow the incidence of obesity as well as develop new dietary models that may lead to better patient adherence for long term weight loss success (Dailey et al., 2019).

### Obesity and the Microbiome

Recent evidence demonstrates a possible connection between obesity and obesity-associated abnormalities with gut microbiome (Muscogiuri et al., 2019). Along with increases in diet-derived energy production, it has been well studied that gut bacteria contributes to fatty acid regulation within tissues as well as to chronic, low-grade inflammation, a classic hallmark of obesity (Ejtahed et al., 2016). Low-grade inflammation or chronic inflammation is characterized by a subtle, smaller immune response by the body to noxious stimuli consistently over a longer period of time that can last months or years instead of an acutely large spike in immune activity lasting only a few days to weeks as seen in many pathologic infections. As such, chronic systemic inflammation is greatly exacerbated when animals are fed a high fat diet. It has been reported that high fat diet causes a shift in the balance of gut microbiota composition toward one that is high in unfavorable bacteria species that may predispose an individual to weight gain (Cani et al., 2007). The high degree of inflammation is triggered by LPS a potent endotoxin produced by Gram-negative bacteria whose levels correlate with consumption of a high fat diet (Ejtahed et al., 2016). The intestinal barrier becomes vulnerable and “leaky” as the intestinal tight junctions become more permeable due to the local inflammation. This results in the uptake of LPS and subsequent binding to CD14 receptors located on immune cells such as monocytes and macrophages as well as gut epithelial cells, triggering a systemic pro-inflammatory cascade and increased insulin resistance/obesity (Cani and Delzenne, 2009). When the immune cells are triggered to produce an immune response, they release cytokines that can impair insulin signaling and lead to a state of insulin resistance over time. If the systemic inflammation is allowed to progress over an extended period of time, it has the potential to propagate into a chronic inflammatory state. A chronic inflammatory state produces changes in the host’s glucose homeostasis and can further increase caloric intake with subsequent weight gain (Soto et al., 2018). For example, a 2-year adherence to a Mediterranean diet (more nutritionally balanced and lower in dietary fat) can influence the physiology of the gut bacteria positively resulting in lower insulin resistance (Haro et al., 2017) in diabetic patients. This speaks to the critical role of diet in shaping microbiota composition and normalize gut bacterial functionality (Cani and Delzenne, 2009). Conversely, administration of specific bacterial strains has been implicated in adipogenesis, a process that is increased in obesity when excess energy is converted into adipose tissue stores (Woting and Blaut, 2016). During periods of increased consumption of high fat foods, fatty acids are esterified into triglycerides and stored within the adipocytes until the body’s energy usage exceeds its consumption (Voshol et al., 2009). The process of adipogenesis is stimulated by lipoprotein lipase (LPL) that hydrolyzes triglycerides, allowing for their uptake in adipose tissue. White adipose tissue also expresses ANGPTL4, a glycoprotein and LPL inhibitor expressed during periods of fasting (Kersten et al., 2000; Yoon et al., 2000) that decreases triglyceride storage and increases circulating plasma triglyceride levels (Lichtenstein and Kersten, 2010). Mice models expressing high levels of ANGPTL4 are much leaner than mice who have under-expressed ANGPTL4 (Soto et al., 2018). Certain gut bacteria can influence ANGPTL4 levels (Woting and Blaut, 2016). For example, when *Lactobacillus paracasei* was given to germ-free mice, it increased ANGPTL4 expression (Woting and Blaut, 2016) while other strains have shown to decrease its levels (Backhed et al., 2004). Despite the few studies linking the gut microbiome to ANGPTL4 expression, there is currently insufficient evidence to fully support this mechanistic pathway (Blaut and Klaus, 2012).

Similarly, certain bacteria extract energy in the form of calories from specific monosaccharides-ingested food via specialized proteins (Turnbaugh et al., 2006) that can directly impact the host’s weight and metabolic energy balance (Lichtenstein and Kersten, 2010). For example, the carbohydrate response element-binding protein (ChREBP), also known as the central metabolic regulator, is involved in sucrose and fructose metabolism. This protein aids in the conversion of molecules of sucrose and fructose into usable caloric energy in the body. Dysregulations of the ChREBP metabolic pathways including enzymes and transporters implicated in glucose and fructose metabolism may result in the accumulation of undigested sucrose and fructose and subsequent changes in the gut microbiota composition (Ortega-Prieto and Postic, 2019). When sugar metabolism cannot ensue, the extracted energy cannot be provided to the gut microbiome leading to dysregulation in gut microbiota composition and subsequent pathogenicity. Likewise, the liver sterol response element binding protein type-1 (SREBP-1), a transcriptional regulator of lipogenic gene expression is linked to gut bacteria and is involved in bacteria-induced lipogenesis, and metabolic remodeling of adipose tissue, which further propagates the storage of adipose tissue (Kersten et al., 2000; Yoon et al., 2000). When this protein is not adequately functioning due to disruption in the gut microbiome, adipose tissue has a higher propensity to store fat and promote obesity. Indeed, treatment with, specific bacteria strains such as *Akkermansia muciniphila*, probiotics or prebiotics has been shown to downregulate high-fat diet or high cholesterol-induced SREBP-1c expression in the liver (Kobayashi et al., 2018). This was due to decreased translocation of pathogen-associated molecular patterns (Kim et al., 2020) or downregulation of fasting-induced adipose factor (FIA) by enteric bacteria (Leung et al., 2016). Taken together, these findings suggest that metabolic imbalance seen in obesity could possibly be linked to the nutritional manipulation of these energy-extracting, lipogenic bacteria and their respective proteins (Ejtahed et al., 2016).

When observing the role of the microbiota in obesity, the production of SCFA by commensal bacteria cannot go unrecognized. Propionate, butyrate, and acetate are byproducts of indigestible carbohydrate fermentation by commensal bacteria in the proximal colon and the cecum (de Clercq et al., 2016). Several studies have linked elevated SCFA levels with increased BMI, possibly due to the increased SCFA producing species leading to an increase in energy absorption (Okubo et al., 2018). Furthermore, SCFAs have been shown to regulate appetite and induce lipid accumulation in adipose tissue (Fernandes et al., 2014; Goffredo et al., 2016). Although the energy extraction model of SCFAs contradicts the anti-obesity effects of SCFA seen in animals, SCFA role in weight gain has been attributed to a developed desensitization against the appetite suppressing/metabolic effects of SCFA (Ortega-Prieto and Postic, 2019). Once absorbed, the SCFA enter the hepatic portal circulation where they act as substrates for gluconeogenesis and *de novo* lipogenesis within hepatocytes ultimately leading to increased energy production and storage (Ortega-Prieto and Postic, 2019). For example, butyrate, which acts as a ligand for the G-protein coupled receptor-41 (Gpr41), present in enteroendocrine cells is implicated in the secretion of glucagon-like peptide-1 (GLP-1), a gut peptide known to affect host satiety, food transit time, caloric intake and post-prandial glucose/fat metabolism (Wichmann et al., 2013). Indeed, conventionally raised Gpr41 −/− mice are much leaner than their wild type siblings (Huang and Hu, 2015), revealing a correlation between improved metabolism, satiety and Gpr41 stimulation by butyrate produced by commensal bacteria in the host gut (Huang and Hu, 2015). By contrast, increased fecal levels of propionate have been observed in obese individuals, whereas this has not been the case for their lean counterparts (Schwiertz et al., 2010; Hartstra et al., 2015). While some studies show elevated fecal SCFA in obese individuals, others indicate that SCFA treatment decreases adiposity and stimulates weight loss (Ley et al., 2006). A key point noted in these studies is that fecal SCFA levels do not directly relate to the actual metabolism of SCFA. Therefore, SCFA most likely elicit satiating, anti-obesity effects in individuals who are in a non-hypercaloric state. However, overexposure to SCFA can cause a desensitizing effect that often results in weight gain and obesity due to hepatic gluconeogenesis and *de novo* lipogenesis.

### Microbiome Composition and Obesity

The lack of diversity in the gut microbiome is commonly associated with obese individuals. Subjects with low microbial gene richness (MGR) often exhibit chronic inflammation, poor insulin sensitivity and higher BMIs (Cotillard et al., 2013; Le Chatelier et al., 2013). However, when these obese subjects consume semi-restrictive, fiber-rich diets, their MGRs drastically increase (Aron-Wisnewsky et al., 2019). Similarly, when morbidly obese individuals underwent Roux-en-Y gastric bypass (RYGB) surgery, they not only lost significant amount of weight, their MGR also increased along with their overall basal metabolic rate (Kong et al., 2013). The drastic changes in microbiome composition observed within the first week post-surgery consisted of increases in *Bacteroides*, *Verrucomicrobia*, and *Proteobacteria* species, all of which have been associated with overall metabolic improvements, better glucose handling, decreased triglyceride levels and weight loss (Chen and Devaraj, 2018). Some specific bacterial strains have been closely associated with obesity while others were inversely correlated. Anti-obesity characteristics attributed to microbiota composition have been associated with fiber utilization and modulation by the microbiome (Santos et al., 2019). It has been documented that soluble fiber consumption is closely linked with delayed gastric emptying and less glucose uptake while insoluble fiber intake has demonstrated early satiety effects, alterations in gut motility and gut hormone secretions (Santos et al., 2019). These attributes favor a leaner body habitus, less metabolic derangements and reduction in the development of chronic pathologies like cancer and cardiovascular disease (Santos et al., 2019). *A. muciniphila* is one such species commonly linked to anti-obesity characteristics (Okubo et al., 2018). For example, in mice and few human studies, *A. muciniphila* significantly improved body composition as well as nutrient handling in obese subjects (Okubo et al., 2018). *A. muciniphila* was less represented in the microbiome of type-2 diabetic mice models (Okubo et al., 2018). Conversely, there was a greater abundance of *Firmicutes* in obese individuals compared to their lean counterparts in several human trials (Wichmann et al., 2013). The *Firmicutes* to *Bacteroidetes* ratio has been used frequently when associating changes in the microbiota composition with an obese phenotype. It has been documented that obese subjects have a higher prevalence of *Firmicutes* in comparison to *Bacteroidetes* species while leaner individuals have many more *Bacteroidetes* and fewer *Firmicutes* (Furet et al., 2010; Louis et al., 2016). When subjects where fed energy-rich diets, *Firmicutes* drastically increased along with the body weight (as expected in a hypercaloric state) (Woting and Blaut, 2016). The mechanism by which this bacterial shift occurs appears to be due to the improved caloric extraction of food by the gut (Le Chatelier et al., 2013). More specifically, increases in glycoside hydrolase as well as ATP binding cassettes were upregulated in mice models expressing more *Firmicutes* (Le Chatelier et al., 2013). However, whether increased *Firmicutes* was actually the catalyst for the observed weight gain directly, remains to be seen. The beneficial effects of an ample *Bacteroides* load displayed in lean individuals, has been attributed to enhanced polysaccharide digesting capabilities with better nutrient metabolism (Chang et al., 2019). However, these findings have not been consistent, as other studies using animal models have demonstrated decreases in *Bacteroidetes* with no change in *Firmicutes*, as well as other changes in the bacteria representation, showing no change in metabolic functionality or polysaccharide digesting capabilities (Ejtahed et al., 2016). These findings may question the previously attributed importance of the *Firmicutes* to *Bacteroides* ratio in human obesity (Goffredo et al., 2016) and suggest a need for further work and evaluation of prior results.

### FMT’s Impact on Obesity

Potential treatments for obesity have been intensely studied over the past decade. Using fecal microbiome transplants (FMTs) as a means to alter the host’s metabolism has been one of the most recently suggested approach for non-pharmacological, non-invasive therapeutic intervention. The reason behind such treatment stems from several findings. First, when obese and lean subjects were fed an isocaloric diet, the obese individuals stored more calories as fat compared to their lean counterparts (Jumpertz et al., 2011). Second, fecal microbiota transfer from obese donor to a lean recipient recapitulated the obese phenotype as well as associated metabolic dysfunctions. Therefore, it is reasonable to assume that the reverse may be true, i.e., microbiota transfer from a lean individual would result in decreased adiposity (Ridaura et al., 2013). This rationale formed the basis for studies examining the relationship between the microbiome and obesity with the implication of FMT as a potential treatment option. As such, the majority of studies involving fecal matter transfer are conducted in animal models. In the now well-known classical work, Backhed et al. (2004) showed a staggering 57% increase in total body fat in germ free (GF) mice inoculated with fecal transplants from conventionally raised siblings (with higher body fat) (Kersten et al., 2000). This acute increase in body fat observed in the GF mice was originally attributed to an increase in energy harvesting by the transplanted bacteria from their conventionally raised, overweight siblings (Han and Lin, 2014). Numerous other animal studies that followed yielded similar results. They also showed reduced intestinal permeability, increased appetite with subsequent hyperphagia, increased adipogenesis and increased intestinal inflammation (Grigorescu and Dumitrascu, 2016). Many of these metabolic changes occurred when caloric intake was held constant between the test groups (Louis et al., 2016). One study looked at the effect of GLP-1, an anorexigenic hormone and bile acids in mice after FMT (Allegretti et al., 2019). It was determined that, when compared to a placebo group, the FMT group had no change in GLP-1 or bile acid synthesis but did show sustained alterations in their microbiota profile (Jumpertz et al., 2011). The actual BMI, however, remained unaltered in the transplanted subjects after 12 weeks of observation (Jumpertz et al., 2011). The conclusion from the study was that the FMT treatment did alter the composition of gut flora but it had no effect on the actual BMI of the test subjects (Jumpertz et al., 2011). Conversely, it has been documented that *A. muciniphila* and direct inoculation of SCFAs have a role in the upregulation of intestinal L-cells that are responsible for the synthesis of GLP-2 and PYY, both pro-satiety peptides (Covasa et al., 2019). Similarly, Duca et al. (2012) concluded that when germ-free mice lacking a gut microbiome were given 48-h access to intralipid emulsions, they consumed more lipid than their control counterparts. In addition, germ free mice had lower levels of GLP-1, PYY, CCK, and ghrelin and lower levels of the peptide leptin. Results from human studies related to the effect of FMT on weight gain come primarily from work where FMT was employed in *C. difficile* treatment. These serendipitous observations showed that lean subjects suffering from *C. difficile* and treated with FMT experienced unexpected weight gain (Turnbaugh et al., 2006). After further investigation it was discovered that their fecal donors were obese individuals. This led to the hypothesis that fecal transplants from obese to lean individuals may result in increased BMI (Turnbaugh et al., 2006). However, direct human clinical trials demonstrating the efficacy of FMT in weight loss are scarce. To date, there is no significant proof that fecal transplants can induce fat accrual in lean individuals or cause adipose tissue reduction in the obese (Aron-Wisnewsky et al., 2019). The use of FMT in the treatment of obesity is still in it’s infancy and large-scale, human studies are needed before more concrete conclusions can be ascertained.

## FMT: Metabolic Syndrome and Type 2 Diabetes

Since early 80’s, the prevalence of type-2 diabetes mellitus (T2DM) has increased significantly with no signs of abating. According to the recent prognosis of the International Federation for Diabetes there are 425 million people worldwide diagnosed with diabetes and that number is expected to rise to 692 million by 2045 (Han and Lin, 2014). Today’s 425 million represents nearly three-fold increase in the number of diabetic patients since 1980 (Fernandes et al., 2014). Metabolic syndrome is a disorder characterized by dyslipidemia, excess body fat levels, hypertension and hyperglycemia (Fernandes et al., 2014). If left untreated, metabolic syndrome, can lead to T2DM, heart disease, atherosclerosis, non-alcoholic fatty liver disease, neuropathy, retinopathy, nephropathy as well as stroke and other pathologic complications (Leustean et al., 2018). T2DM, is characterized by the underproduction of insulin by pancreatic beta cells and increase in resistance to insulin stimulation perpetuating uncontrolled glucose homeostasis (Leustean et al., 2018). Recent trends of poor nutrition coupled with obesity related excess caloric consumption has led to the drastic increase in metabolic syndrome related diseases, such as T2DM (Tilg and Kaser, 2011; Palermo et al., 2014). A genetic component has also been identified as a contributing factor to disease development and progression (Ussar et al., 2015). The multifactorial interaction between diet and the genome demonstrated in metabolic syndrome/T2DM helps explain the broad spectrum of phenotypic variation amongst obese individuals (Allegretti et al., 2019).

### Metabolic Syndrome and the Microbiome

Metabolic syndrome, and related disorders, are characterized by a high degree of inflammation (Han and Lin, 2014). This inflammatory process is underlined by systemic activation of proinflammatory cytokines, along with inflammation-propagating signal transduction pathways by immune cells such as CD8+ T cells and Th1 cells (Hotamisligil, 2006; Esser et al., 2014). Chronic inflammation ultimately leads to insulin resistance and metabolic syndrome as a result of the uninterrupted cytokine release damaging the insulin-sensitive cells in liver, muscle and adipose tissue. Studies have shown that when there is a genetic deficiency in pro-inflammatory immune cells such as CD8+ T cells or CD4+ Th1 cells insulin sensitivity is improved while the opposite effect is observed when mouse models were transferred CD8+ T cell/CD4+ Th1 cells (Wu and Ballantyne, 2020). In the GI tract, the initial induction of the inflammatory cascade and cytokine release is triggered by the binding of LPS to toll-like receptor 4 (TLR4) on various immune cells such as macrophages and CD8+ T cells (Leustean et al., 2018). The gut flora have a protective barrier (the intestinal mucosal membrane), which is vital in the resistance of endotoxin-related pathogen infection and subsequent inflammation (Okubo et al., 2018). When this barrier is damaged by cytokine-induced inflammation, metabolic derangement is observed. Similarly, it has been documented that when this barrier is breached as seen in metabolic disease and obese individuals, anti-IgG antibodies against commensal bacteria are induced to cause further systemic inflammation and damage metabolic processes (Tilg et al., 2020). In addition, certain strains of bacteria such as *Ruminococcus gnavus* and *Bacteroides* species, which commonly inhabit the gut, are considered “pro-inflammatory” in nature and can further contribute to the chronic inflammatory state seen in metabolic disease and obesity (Tilg et al., 2020). These specific pro-inflammatory strains are of great abundance in individuals with high BMI and those with metabolic disease (Tilg et al., 2020). A chronic inflammatory state is a key hallmark observed in metabolic syndrome, obesity and related diseases due to its propensity to induce a state of insulin resistance (Leustean et al., 2018).

### Gut Disequilibrium and Diabetes

As in the case with obesity, several commensal bacterial species have been associated with T2DM. More specifically, gut bacterial imbalance has been identified as a modulator in the autoimmune destruction of pancreatic beta cells demonstrated in type 2 diabetics (Louis et al., 2016). A prime candidate, *A. muciniphila*, an intestinal mucin-degrading Gram-negative bacterium, with anti-inflammatory effects that is involved in gut mucosal barrier functionality, has been associated with metabolic syndrome related pathologies. As such, diabetic patients have less *A. muciniphila* in comparison to healthy individuals (Zhang et al., 2019). They also exhibit a greater susceptibility to pathogenic infection and inflammatory stimulation due to a hyperpermeable, vulnerable intestinal mucosal layer. Similarly, T2DM patients not taking diabetic-related drugs have fewer *Firmicutes* when compared to the non-T2DM controls (Larsen et al., 2010), although both groups display similar total levels of bacterial abundance (Tilg and Kaser, 2011). Furthermore, when comparing women with T2DM, to those with moderate glucose intolerance and healthy controls, women with T2DM had a considerable assortment of *Lactobacillus* in comparison to both groups with impaired glucose tolerance and the healthy control (Karlsson et al., 2013). Along with changes in the abundance of Lactobacilli, T2DM individuals had much lower levels of Clostridium (Ussar et al., 2015). *Lactobacillus* has been associated with elevated fasting blood glucose and HbA1c levels when in non-physiologic numbers while *Clostridium* has displayed opposite effects (Le Chatelier et al., 2013). Clostridium have also been linked to butyrate production, an anti-obesity compound (Le Chatelier et al., 2013). Moreover, patients with T2DM have decreased levels of both *Roseburia* and *Faecalibacterium prausnitzii* (from the phylum *Firmicutes*), which are also involved in the production of butyrate when compared to non-diabetics (Ussar et al., 2015). Increased levels of *F. prausnitzii* has also been correlated with decreased inflammation, obesity and metabolic syndrome when calories are held constant, even in patients with T2DM (Louis et al., 2016). Although these results have yet to be reproduced, they can potentially serve as reference diagnostic identifiers for T2DM in high-risk individuals (Qin et al., 2012). The lower levels of butyrate producing species like *F. prausnitzii*, *Roseburia*, and *Clostridium* demonstrated in individuals with T2DM suggests a possible link between butyrate production and T2DM manifestation (Biagi et al., 2010; Hur and Lee, 2015).

Similarly, there has also been a link between T2DM medications and the gut microbiota. A common drug of choice for treating T2DM has been metformin. Metformin works by inhibiting gluconeogenesis in the liver as a means to lower blood glucose levels and increase cellular glucose uptake as a result of improved insulin sensitivity (Okubo et al., 2018). Additionally, metformin has been shown to upregulate *Escherichia* and *A. muciniphila* in the gut, both of which are involved in butyrate production (Okubo et al., 2018). However, the consequential increase in *A. muciniphila* is not directly correlated with a decreased HbA1c even though better glucose control has been observed (Okubo et al., 2018). Similarly, when the gut microbiome of metformin-treated mice was transplanted into the GI tracts of germ-free mice, a substantial improvement in glucose handling was observed (Okubo et al., 2018). Other studies have shown increases in *Bacteroides*, *Butyricimonas*, and *Parabacteroides* that have been known to decrease IL-1 and IL-6 in epididymal fat. IL-1 and IL-6 are both key interleukins released during the inflammatory cascade, an effect that has been seen in response to metformin treatment (Lee et al., 2018). This data show a similarity between the effects of bacteria such *Akkermansia* and of metformin as they both seem to lower tissue inflammation that is commonly observed in diet-induced obesity (Zhang et al., 2019). Further trials are no doubt necessary in order to mimic the effects of glucose-lowering drugs like metformin through alternative therapeutic approaches such as the development of specific prebiotics, probiotics, or symbiotics.

The extent to which gut microbiota is involved in metabolic parameters associated with T2DM and at what stage is not entirely known. It has been suggested that gut bacteria may play a more significant role in the earlier stages of glycemic control and to a lesser degree in severe stages of insulin resistance observed in advanced T2DM (Aron-Wisnewsky et al., 2019). Some proposed mechanisms for gut microbiota involvement in early T2DM include SCFA regulation, adipose tissue associated inflammation and bile acid compositional changes (Aron-Wisnewsky et al., 2019). Although the link between the gut microbiome and metabolic syndrome has been well documented in the scientific literature, further research is needed in order to decipher the relationship involving bacterial disequilibrium and metabolic syndrome.

### Metabolic Syndrome, Diabetes and FMT

The concept of using FMT in the treatment of metabolic syndrome and T2DM is complex. As previously mentioned, a key pathway involved in the development of insulin resistance that occurs in T2DM and metabolic syndrome is the activation of the TLR on immune cells causing the inflammation characteristic of insulin resistance. Specifically, TLR4, an extracellular cell surface receptor expressed in immune cells and enterocytes, is involved in the desensitization of insulin stimulation. TLR4 activation dampens the effects of insulin through the activation of various pro-inflammatory mediators by triggering signaling cascades and transcriptional factors such as MyD88, TIRAP, TRIF, IKKs, and JNKs involved in the innate immune responses and ultimately leads to the development of insulin resistance (Le Chatelier et al., 2013). A deficit in these TLRs results in increased susceptibility to cellular damage (Le Chatelier et al., 2013). TLR4 has a protective immune response against bacterial invasion. As such, overexpressing the TLR4 signaling pathways results in increased bacterial density in the colonic mucosa and increased bacterial translocation (Dheer et al., 2016). Conversely, the lack of TLR4 (TLR4 knock-out) reduces the abundance of *Bacteroides* and *Alloprevotella* which can strongly affect the host immune system (Xiao et al., 2019). Ligands to TLRs include components of the Gram-positive and Gram-negative bacteria which can cause disruptions to TLR-mediated pathways resulting in lack of protection against gut injury. For instance, when the microbiota of TLR5-deficient mice was transplanted into wild-type TLR5 mice, the alteration of the wild type mice’s gut bacterial composition led to the development of obesity, insulin resistance, and metabolic syndrome (Le Chatelier et al., 2013). This was reportedly due to higher levels of Firmicutes and lower levels of Actinobacteria (Le Chatelier et al., 2013). After treatment with antibiotics, the consequential effects of the FMT on their gut were reversed (Le Chatelier et al., 2013). It has been reported that when TLRs are diminished, there is an increased preponderance of diabetic nephropathy as well as other diabetic-related conditions (Dasu et al., 2010; Caricilli et al., 2011). Likewise, when germ-free mice were inoculated with fecal transplants from metformin-treated mice donors, they experienced improved metabolic and anti-inflammatory effects (Lee et al., 2018). This is primarily due to an improved lipid profile and decreased body weight in the treated mice (Lee et al., 2018). Most individuals diagnosed with T2DM take drug that lowers glucose, such as metformin, which have been shown to alter the microbiome composition. This pharmacological intervention may obscure and interfere with the transplantee’s engraftment of the host’s bacteria and could mask the potential therapeutic effects of the transplant (Aron-Wisnewsky et al., 2019). The effect of FMT on modulation of gut microbiota in T2DM was recently demonstrated in *db/db* mice using a modified microbiota following treatment with *Sennoside A*, a glucoside present in rhubarb shown to reduce blood glucose, increase intestinal barrier integrity, decrease LPS translocation, tissue inflammation and insulin resistance. As such, fecal transfer from *Sennoside A*-treated mice decreased blood glucose levels, insulin resistance and body weight in *db/db* mice. These changes were associated with significant changes in the relative abundance of *Akkermansia*, *Muscispirillum*, *Oscillospira*, and *Ruminococcus*. Taken together, these results show that a “metabolically favorable” microbiota profile can be modeled and used successfully to restore a “metabolically unfavorable” microbiota profile in correcting metabolic disorders (Wei et al., 2020).

**FIGURE 2:**
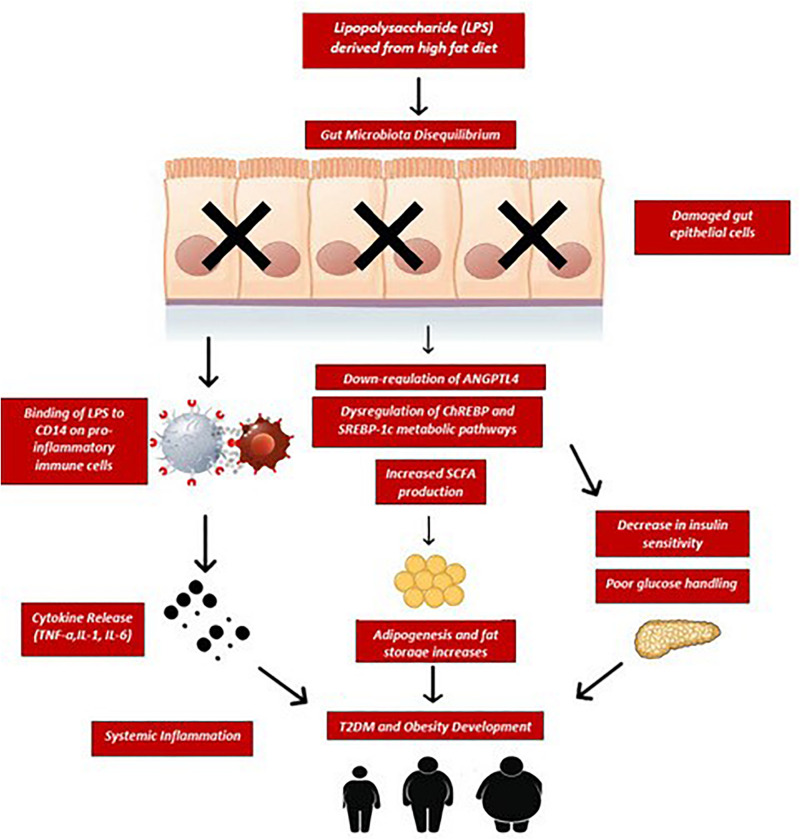
Sustained consumption of a high fat diet elevates bacterial lipopolysaccharides (LPS) entering circulation as a result of disequilibrium in gut microbiota composition and increased intestinal permeability. LPS binds to CD14 receptors on gut epithelial and immune cells which trigger the release of pro-inflammatory cytokines such as TNF-α, IL-1, and IL-6. These cytokines induce a state of systemic low-grade inflammation and cause further damage to the epithelial barrier, resulting in metabolic endotoxemia. Similarly, fecal SCFA overproduction has been associated with increased adipogenesis, excess lipid accumulation in fat stores and subsequent risks for obesity and diabetes in humans. Furthermore, this chronic inflammatory state causes dysregulation of ChREBP and SREBP-1c transcription factors as well as downregulation of ANGPTL4 leading to increased insulin resistance and impaired glucose tolerance. If this chronic inflammatory state, caloric overfeeding and poor glucose handling remains unopposed, T2DM and obesity may develop. ChREBP, carbohydrate-response element-binding protein; SREBP-1c, sterol regulatory element-binding protein 1c; ANGPTL4, angiopoietin-like 4; CD14, cluster of differentiation-14; TNF-α, tumor necrosis factor; IL-1, interleukin-1; IL-6, interleukin-6; SCFA, short chain fatty acids.

In humans, there has only been one reported study that has tested the effects of FMT in patients with T2DM without the presence of anti-diabetic medications. In a randomized control trial testing FMT in men with T2DM, A. Vrieze et al. (2012) found that when subjects received duodenal tube-facilitated fecal transplants from lean individuals (allogenic transplantation) their insulin sensitivity improved in concordance with increased microbial diversity compared to individuals who received autologous transplantation (stool from self) after a 6 week period. Among changes in microbial composition, the increase in *Roseburia intestinalis* and *Eubacterium hallii*, both of which are butyrate-producing organisms, was the most notable (Hur and Lee, 2015). There were no changes in BMI during the 6-week period after the fecal transplantation (Marotz and Zarrinpar, 2016). It is worth noting that not all participants responded to the FMT and the study, as a whole, included a modest number of subjects (*n* = 18) (Vallianou et al., 2018). Therefore, the existing evidence precludes us to conclude with certainty that FMT is warranted in patients suffering from T2DM and metabolic syndrome (Vallianou et al., 2018). However, the few studies showing a beneficial effect are promising and more work might shed additional light on the benefits of FMT as a valid option for patients with T2DM and metabolic syndrome in the future. Many of the observed physiological changes post-FMT are anti-diabetic in nature including improved glucose handling, basal metabolic rate enhancement and lower levels of systemic inflammation (Figure 2).

## Perspectives

Thus far, applications of FMT have been limited to the treatment of *C. difficile* infections where fecal transplant has proved highly efficacious. On the other hand, studies using FMT to treat other disorders that are related to gut dysbiosis, have yielded mixed conclusions. Several studies show beneficial effects of FMT application such as improved glucose handling, decreased systemic inflammation and basal metabolic rate improvements while others showed little to no clinical significant effects. More research examining the role of FMT in the treatment of obesity, T2DM and metabolic syndrome is needed in order to better understand its therapeutic properties in modern medicine. Although the concept of using FMT has been around for some time, the impact of FMT on specific disease processes has not kept pace with recent progress in quantitative metagenomics. This is not surprising as substantial amount of work has been dedicated toward identifying and characterizing the role of various bacteria both in health and disease. Notwithstanding the significant advances in the past two decades in quantitative metagenomics, our understanding of the functions of bacteria and their metabolic by-products remain scarce.

In addition to examining the role of FMT in metabolic disorders, studies should also focus on testing the efficacy of FMT in other disease processes that have similar pathogenicity as metabolic syndrome including allergen-related GI disease like Celiac disease. The pathophysiology of Celiac Disease involves autoimmune destruction of GI epithelial cells due to the influx of specific gluten-related peptides such as gliadin (Parzanese et al., 2017). Specifically, ingestion of gluten-containing products such as wheat, barley and rye, triggers a T-cell-mediated inflammatory cascade leading to villous atrophy, intestinal crypt hyperplasia and chronic malabsorption within the gut (Vrieze et al., 2012). Both Celiac Disease and metabolic derangements can be attributed to the epithelial destruction caused by chronic inflammatory states and a pronounced dysbiotic microbial environment. One documented feature of the gut microbiome is providing stability and integrity of the epithelial lining of the gut. When the epithelial barrier is damaged like in Celiac Disease, the barrier becomes permeable to various antigens such as gliadin which triggers the inflammatory immune response and subsequent sequala of Celiac-related pathogenesis. Restoration of the so called “disrupted” microbiota through fecal transplant could potentially help prevent the gliadin from leaking through the epithelial barrier and lessen the inflammation-induced destruction of the villi, allowing for better nutrient absorption in patients suffering from Celiac Disease. This is not without precedent, since FMT has been proven successful in curing infection with an antibiotic-resistant strain of *C. difficile*. This is an example of how remodeling of a severely disrupted gut microbial configuration by *C. difficile infection* can mitigate its debilitating clinical symptomatology. How the transplanted “whole-gut” bacteria adapt and function in the newly host environment to affect phenotypic changes is not completely known and is challenging to quantify (Seddon et al., 2014). Studies using a more targeted approach where one or selected few known bacteria strains are inoculated in a new host can provide better insights into their role and interactions leading to a successful restoration of microbiota composition. This complex relationship is compounded by numerous host variables such as individual conditions, disease status, dietary habits, development, physiological status, gut microbial composition, and other. In this respect, more work is needed to answer questions about not only which bacteria strains or species are the most effective in altering a particular gut microbiota phenotype, but also how many species or strains are required to restore a severely damaged gut ecosystem. Since effective colonization using FMT depends on the microbiota profile of the recipient that vary between metabolic syndrome patients (Li et al., 2016), the selection of bacteria based on their resilience, specificity and ability to compete within an existent microbial ecosystem is a key step in obtaining the desired clinical outcome. Of critical importance are the findings demonstrating associations between specific bacterial species or strains and improvements in health outcomes. Therefore, the goal of the FMT is to re-optimize the dysbiotic gut environment and prevent further development of the disease. To do this, some authors recommend targeted approaches that take into account the host’s genetic, physiological and metabolic profile to achieve the desired microbial diversity. Furthermore, the gut microbiota is also susceptible to the external environment brought about by the industrialized way of modern living. In this context, engineering a customized consortia of bacteria designed to restore a certain disease microbial profile through FMT might represent future remedy as key in maintaining a healthy gut microbiota. The identification of specific microbial consortia that are associated with certain diseases coupled with information on the host’s genomic and metabolic signatures may help in personalizing a healthy gut ecosystem. Although there are still many unknowns surrounding FMT and the gut microbiome, new discoveries will continue to bridge these gaps in our understanding of how FMT can be used effectively to improve metabolic disorders related to gut dysbiosis like obesity and diabetes.

## Conclusion

The gut microbiome is a key modulator of many physiological and metabolic processes including nutrients and drug substances, immunity, inflammation and many diseases. The underlying mechanisms by which gut microbiota affect metabolic syndrome including insulin resistance, dyslipidemia and hypertension are not entirely clear. Among them, the role of SCFA, bile acids, incretin hormones, endotoxins and direct bacterial translocation have been proposed. Chronic inflammatory states have also been linked to gut-related dysfunctions involving microbiome imbalance in obese and diabetic individuals. Even though our understanding of how the gut microbiota is positively impacted by the use of FMT is gradually emerging, we are still far from knowing with high degree of certainty whether or not this procedure is efficacious in the treatment of anything besides chronic *C. difficile infection*. In this respect, several pathologies such as obesity, metabolic syndrome, irritable bowel syndrome, enterocolitis and intra-intestinal disorders have been characterized by an imbalanced gut microbiota. Despite the results from few human studies where the restoration of beneficial gut bacteria through the use of FMT has led to promising results, currently they do not fully support the use of fecal microbial transplants in the treatment of metabolic diseases (Falony et al., 2019). With the potential for negative side effects, the use of FMT for conditions other than recurrent *C. difficile* infections is ill-advised at this current time. However, the evidence from studies showing remedial effects such as weight loss and better glycemic control are promising and beg the need for further research into the role of FMT in the pathophysiology of metabolic syndrome.

## Author Contributions

Both authors wrote and revised the manuscript.

## Conflict of Interest

The authors declare that the research was conducted in the absence of any commercial or financial relationships that could be construed as a potential conflict of interest.

## References

[B1] Allegretti J. R. Kassam Z. Mullish B. H. Chiang A. Carrellas M. Hurtado J. (2019). Effects of fecal microbiota transplantation with oral capsules in obese patients. *Clin. Gastroenterol. Hepatol*. 18 855.e2–863.e2. 31301451 10.1016/j.cgh.2019.07.006

[B2] Aron-Wisnewsky J. Clement K. Nieuwdorp M. (2019). Fecal microbiota transplantation: a future therapeutic option for obesity/diabetes? *Curr. Diab. Rep.* 19:51. 10.1007/s11892-019-1180-z 31250122

[B3] Backhed F. Ding H. Wang T. Hooper L. V. Koh G. Y. Nagy A. (2004). The gut microbiota as an environmental factor that regulates fat storage. *Proc. Natl. Acad. Sci. U.S.A.* 101 15718–15723. 10.1073/pnas.0407076101 15505215 PMC524219

[B4] Benson A. K. Kelly S. A. Legge R. Ma F. Low S. J. Kim J. (2010). Individuality in gut microbiota composition is a complex polygenic trait shaped by multiple environmental and host genetic factors. *Proc. Natl. Acad. Sci. U.S.A.* 107 18933–18938. 10.1073/pnas.1007028107 20937875 PMC2973891

[B5] Biagi E. Nylund L. Candela M. Ostan R. Bucci L. Pini E. (2010). Through ageing, and beyond: gut microbiota and inflammatory status in seniors and centenarians. *PLoS One* 5:e10667. 10.1371/journal.pone.0010667 20498852 PMC2871786

[B6] Blaut M. Klaus S. (2012). Intestinal microbiota and obesity. *Handb. Exp. Pharmacol.* 209 251–273. 10.1007/978-3-642-24716-3_11 22249818

[B7] Cani P. D. Amar J. Iglesias M. A. Poggi M. Knauf C. Bastelica D. (2007). Metabolic endotoxemia initiates obesity and insulin resistance. *Diabetes* 56 1761–1772. 17456850 10.2337/db06-1491

[B8] Cani P. D. Delzenne N. M. (2009). The role of the gut microbiota in energy metabolism and metabolic disease. *Curr. Pharm. Des.* 15 1546–1558. 10.2174/138161209788168164 19442172

[B9] Caricilli A. M. Picardi P. K. De Abreu L. L. Ueno M. Prada P. O. Ropelle E. R. (2011). Gut microbiota is a key modulator of insulin resistance in TLR 2 knockout mice. *PLoS Biol.* 9:e1001212. 10.1371/journal.pbio.1001212 22162948 PMC3232200

[B10] Chang C. S. Ruan J. W. Kao C. Y. (2019). An overview of microbiome based strategies on anti-obesity. *Kaohsiung J. Med. Sci.* 35 7–16. 10.1002/kjm2.12010 30844145 PMC11900707

[B11] Chen X. Devaraj S. (2018). Gut microbiome in obesity, metabolic syndrome, and diabetes. *Curr. Diab. Rep.* 18:129. 10.1007/s11892-018-1104-3 30338410

[B12] Clavel T. Desmarchelier C. Haller D. Gerard P. Rohn S. Lepage P. (2014). Intestinal microbiota in metabolic diseases: from bacterial community structure and functions to species of pathophysiological relevance. *Gut Microbes* 5 544–551. 10.4161/gmic.29331 25003516

[B13] Cotillard A. Kennedy S. P. Kong L. C. Prifti E. Pons N. Le Chatelier E. (2013). Dietary intervention impact on gut microbial gene richness. *Nature* 500 585–588. 10.1038/nature12480 23985875

[B14] Covasa M. Stephens R. W. Toderean R. Cobuz C. (2019). Intestinal sensing by gut microbiota: targeting gut peptides. *Front. Endocrinol.* 10:82. 10.3389/fendo.2019.00082 30837951 PMC6390476

[B15] Dailey F. E. Turse E. P. Daglilar E. Tahan V. (2019). The dirty aspects of fecal microbiota transplantation: a review of its adverse effects and complications. *Curr. Opin. Pharmacol.* 49 29–33. 10.1016/j.coph.2019.04.008 31103793

[B16] Dasu M. R. Devaraj S. Park S. Jialal I. (2010). Increased toll-like receptor (TLR) activation and TLR ligands in recently diagnosed type 2 diabetic subjects. *Diabetes Care* 33 861–868. 10.2337/dc09-1799 20067962 PMC2845042

[B17] de Clercq N. C. Groen A. K. Romijn J. A. Nieuwdorp M. (2016). Gut microbiota in obesity and undernutrition. *Adv. Nutr.* 7 1080–1089. 10.3945/an.116.012914 28140325 PMC5105041

[B18] DeFilipp Z. Bloom P. P. Torres Soto M. Mansour M. K. Sater M. R. A. Huntley M. H. (2019). Drug-resistant *E. coli* bacteremia transmitted by fecal microbiota transplant. *N. Engl. J. Med.* 381 2043–2050. 10.1056/nejmoa1910437 31665575

[B19] Dheer R. Santaolalla R. Davies J. M. Lang J. K. Phillips M. C. Pastorini C. (2016). Intestinal epithelial toll-like receptor 4 signaling affects epithelial function and colonic microbiota and promotes a risk for transmissible colitis. *Infect. Immun.* 84 798–810. 10.1128/iai.01374-15 26755160 PMC4771346

[B20] Donaldson G. P. Lee S. M. Mazmanian S. K. (2016). Gut biogeography of the bacterial microbiota. *Nat. Rev. Microbiol.* 14 20–32. 10.1038/nrmicro3552 26499895 PMC4837114

[B21] Duca F. A. Swartz T. D. Sakar Y. Covasa M. (2012). Increased oral detection, but decreased intestinal signaling for fats in mice lacking gut microbiota. *PLoS One* 7:e39748. 10.1371/journal.pone.0039748 22768116 PMC3387243

[B22] Ejtahed H. S. Soroush A. R. Angoorani P. Larijani B. Hasani-Ranjbar S. (2016). Gut microbiota as a target in the pathogenesis of metabolic disorders: a new approach to novel therapeutic agents. *Horm. Metab. Res.* 48 349–358. 10.1055/s-0042-107792 27203411

[B23] El-Matary W. Simpson R. Ricketts-Burns N. (2012). Fecal microbiota transplantation: are we opening a can of worms? *Gastroenterology* 143 e19–e20. 10.1053/j.gastro.2012.04.055 22732575

[B24] Esser N. Legrand-Poels S. Piette J. Scheen A. J. Paquot N. (2014). Inflammation as a link between obesity, metabolic syndrome and type 2 diabetes. *Diabetes Res. Clin. Pract.* 105 141–150. 10.1016/j.diabres.2014.04.006 24798950

[B25] Falony G. Vandeputte D. Caenepeel C. Vieira-Silva S. Daryoush T. Vermeire S. (2019). The human microbiome in health and disease: hype or hope. *Acta Clin. Belg.* 74 53–64. 10.1080/17843286.2019.1583782 30810508

[B26] Fernandes J. Su W. Rahat-Rozenbloom S. Wolever T. M. Comelli E. M. (2014). Adiposity, gut microbiota and faecal short chain fatty acids are linked in adult humans. *Nutr. Diabetes* 4:e121. 10.1038/nutd.2014.23 24979150 PMC4079931

[B27] Furet J. P. Kong L. C. Tap J. Poitou C. Basdevant A. Bouillot J. L. (2010). Differential adaptation of human gut microbiota to bariatric surgery-induced weight loss: links with metabolic and low-grade inflammation markers. *Diabetes* 59 3049–3057. 10.2337/db10-0253 20876719 PMC2992765

[B28] Goffredo M. Mass K. Parks E. J. Wagner D. A. Mcclure E. A. Graf J. (2016). Role of gut microbiota and short chain fatty acids in modulating energy harvest and fat partitioning in youth. *J. Clin. Endocrinol. Metab.* 101 4367–4376. 10.1210/jc.2016-1797 27648960 PMC5095239

[B29] Gorvitovskaia A. Holmes S. P. Huse S. M. (2016). Interpreting prevotella and *Bacteroides* as biomarkers of diet and lifestyle. *Microbiome* 4:15. 10.1186/s40168-016-0160-7 PMC4828855 27068581

[B30] Gough E. Shaikh H. Manges A. R. (2011). Systematic review of intestinal microbiota transplantation (fecal bacteriotherapy) for recurrent *Clostridium difficile* infection. *Clin. Infect. Dis.* 53 994–1002. 10.1093/cid/cir632 22002980

[B31] Grehan M. J. Borody T. J. Leis S. M. Campbell J. Mitchell H. Wettstein A. (2010). Durable alteration of the colonic microbiota by the administration of donor fecal flora. *J. Clin. Gastroenterol.* 44 551–561. 10.1097/mcg.0b013e3181e5d06b 20716985

[B32] Grigorescu I. Dumitrascu D. L. (2016). Implication of gut microbiota in diabetes mellitus and obesity. *Acta Endocrinol.* 12 206–214. 10.4183/aeb.2016.206 31149088 PMC6535288

[B33] Gu S. Chen D. Zhang J. N. Lv X. Wang K. Duan L. P. (2013). Bacterial community mapping of the mouse gastrointestinal tract. *PLoS One* 8:e74957. 10.1371/journal.pone.0074957 24116019 PMC3792069

[B34] Gutin L. Piceno Y. Fadrosh D. Lynch K. Zydek M. Kassam Z. (2019). Fecal microbiota transplant for Crohn disease: a study evaluating safety, efficacy, and microbiome profile. *United Eur. Gastroenterol. J.* 7 807–814. 10.1177/2050640619845986 31316785 PMC6620877

[B35] Hamilton M. J. Weingarden A. R. Sadowsky M. J. Khoruts A. (2012). Standardized frozen preparation for transplantation of fecal microbiota for recurrent *Clostridium difficile* infection. *Am. J. Gastroenterol.* 107 761–767. 10.1038/ajg.2011.482 22290405

[B36] Han J. L. Lin H. L. (2014). Intestinal microbiota and type 2 diabetes: from mechanism insights to therapeutic perspective. *World J. Gastroenterol.* 20 17737–17745. 10.3748/wjg.v20.i47.17737 25548472 PMC4273124

[B37] Haro C. Garcia-Carpintero S. Rangel-Zuniga O. A. Alcala-Diaz J. F. Landa B. B. Clemente J. C. (2017). Consumption of two healthy dietary patterns restored microbiota dysbiosis in obese patients with metabolic dysfunction. *Mol. Nutr. Food Res.* 61:1700300. 10.1002/mnfr.201700300 28940737

[B38] Hartstra A. V. Bouter K. E. Backhed F. Nieuwdorp M. (2015). Insights into the role of the microbiome in obesity and type 2 diabetes. *Diabetes Care* 38 159–165. 10.2337/dc14-0769 25538312

[B39] Hotamisligil G. S. (2006). Inflammation and metabolic disorders. *Nature* 444 860–867. 10.1038/nature05485 17167474

[B40] Huang T. Hu F. B. (2015). Gene-environment interactions and obesity: recent developments and future directions. *BMC Med. Genomics* 8(Suppl. 1):S2. 10.1186/1755-8794-8-S1-S2 25951849 PMC4315311

[B41] Human Microbiome Project Consortium (2012). Structure, function and diversity of the healthy human microbiome. *Nature* 486 207–214. 10.1038/nature11234 22699609 PMC3564958

[B42] Hur K. Y. Lee M. S. (2015). Gut microbiota and metabolic disorders. *Diabetes Metab. J.* 39 198–203. 26124989 10.4093/dmj.2015.39.3.198 PMC4483604

[B43] Jayasinghe T. N. Chiavaroli V. Holland D. J. Cutfield W. S. O’sullivan J. M. (2016). The new era of treatment for obesity and metabolic disorders: evidence and expectations for gut microbiome transplantation. *Front. Cell. Infect. Microbiol.* 6:15. 10.3389/fcimb.2016.00015 26925392 PMC4759265

[B44] Jumpertz R. Le D. S. Turnbaugh P. J. Trinidad C. Bogardus C. Gordon J. I. (2011). Energy-balance studies reveal associations between gut microbes, caloric load, and nutrient absorption in humans. *Am. J. Clin. Nutr.* 94 58–65. 10.3945/ajcn.110.010132 21543530 PMC3127503

[B45] Karlsson F. H. Tremaroli V. Nookaew I. Bergstrom G. Behre C. J. Fagerberg B. (2013). Gut metagenome in European women with normal, impaired and diabetic glucose control. *Nature* 498 99–103. 10.1038/nature12198 23719380

[B46] Kersten S. Mandard S. Tan N. S. Escher P. Metzger D. Chambon P. (2000). Characterization of the fasting-induced adipose factor FIAF, a novel peroxisome proliferator-activated receptor target gene. *J. Biol. Chem.* 275 28488–28493. 10.1074/jbc.m004029200 10862772

[B47] Khanna S. Tosh P. K. (2014). A clinician’s primer on the role of the microbiome in human health and disease. *Mayo Clin. Proc.* 89 107–114. 10.1016/j.mayocp.2013.10.011 24388028

[B48] Kim S. Lee Y. Kim Y. Seo Y. Lee H. Ha J. (2020). *Akkermansia muciniphila* prevents fatty liver disease, decreases serum triglycerides, and maintains gut homeostasis. *Appl. Environ. Microbiol.* 86:e3004-19. 10.1128/AEM.03004-19 PMC7082569 31953338

[B49] Kobayashi M. Fujii N. Narita T. Higami Y. (2018). SREBP-1c-Dependent metabolic remodeling of white adipose tissue by caloric restriction. *Int. J. Mol. Sci.* 19:3335. 10.3390/ijms19113335 30373107 PMC6275055

[B50] Kong L. C. Tap J. Aron-Wisnewsky J. Pelloux V. Basdevant A. Bouillot J. L. (2013). Gut microbiota after gastric bypass in human obesity: increased richness and associations of bacterial genera with adipose tissue genes. *Am. J. Clin. Nutr.* 98 16–24. 10.3945/ajcn.113.058743 23719559

[B51] Larsen N. Vogensen F. K. Van Den Berg F. W. Nielsen D. S. Andreasen A. S. Pedersen B. K. (2010). Gut microbiota in human adults with type 2 diabetes differs from non-diabetic adults. *PLoS One* 5:e9085. 10.1371/journal.pone.0009085 20140211 PMC2816710

[B52] Le Chatelier E. Nielsen T. Qin J. Prifti E. Hildebrand F. Falony G. (2013). Richness of human gut microbiome correlates with metabolic markers. *Nature* 500 541–546. 23985870 10.1038/nature12506

[B53] Lee H. Lee Y. Kim J. An J. Lee S. Kong H. (2018). Modulation of the gut microbiota by metformin improves metabolic profiles in aged obese mice. *Gut Microbes* 9 155–165. 10.1080/19490976.2017.1405209 29157127 PMC5989809

[B54] Leung C. Rivera L. Furness J. B. Angus P. W. (2016). The role of the gut microbiota in NAFLD. *Nat. Rev. Gastroenterol. Hepatol.* 13 412–425. 10.1038/nrgastro.2016.85 27273168

[B55] Leustean A. M. Ciocoiu M. Sava A. Costea C. F. Floria M. Tarniceriu C. C. (2018). Implications of the intestinal microbiota in diagnosing the progression of diabetes and the presence of cardiovascular complications. *J. Diabetes Res.* 2018:5205126. 10.1155/2018/5205126 PMC6260408 30539026

[B56] Ley R. E. Turnbaugh P. J. Klein S. Gordon J. I. (2006). Microbial ecology: human gut microbes associated with obesity. *Nature* 444 1022–1023. 17183309 10.1038/4441022a

[B57] Li S. S. Zhu A. Benes V. Costea P. I. Hercog R. Hildebrand F. (2016). Durable coexistence of donor and recipient strains after fecal microbiota transplantation. *Science* 352 586–589. 10.1126/science.aad8852 27126044

[B58] Lichtenstein L. Kersten S. (2010). Modulation of plasma TG lipolysis by Angiopoietin-like proteins and GPIHBP1. *Biochim. Biophys. Acta* 1801 415–420. 10.1016/j.bbalip.2009.12.015 20056168

[B59] Louis S. Tappu R. M. Damms-Machado A. Huson D. H. Bischoff S. C. (2016). Characterization of the gut microbial community of obese patients following a weight-loss intervention using whole metagenome shotgun sequencing. *PLoS One* 11:e0149564. 10.1371/journal.pone.0149564 26919743 PMC4769288

[B60] Lynch J. B. Sonnenburg J. L. (2012). Prioritization of a plant polysaccharide over a mucus carbohydrate is enforced by a *Bacteroides* hybrid two-component system. *Mol. Microbiol.* 85 478–491. 10.1111/j.1365-2958.2012.08123.x 22686399 PMC3404733

[B61] Marotz C. A. Zarrinpar A. (2016). Treating obesity and metabolic syndrome with fecal microbiota transplantation. *Yale J. Biol. Med.* 89 383–388. 27698622 PMC5045147

[B62] Martinez I. Lattimer J. M. Hubach K. L. Case J. A. Yang J. Weber C. G. (2013). Gut microbiome composition is linked to whole grain-induced immunological improvements. *ISME J.* 7 269–280. 10.1038/ismej.2012.104 23038174 PMC3554403

[B63] Muscogiuri G. Cantone E. Cassarano S. Tuccinardi D. Barrea L. Savastano S. (2019). Gut microbiota: a new path to treat obesity. *Int. J. Obes. Suppl.* 9 10–19. 10.1038/s41367-019-0011-7 31391921 PMC6683132

[B64] Okubo H. Nakatsu Y. Kushiyama A. Yamamotoya T. Matsunaga Y. Inoue M. K. (2018). Gut microbiota as a therapeutic target for metabolic disorders. *Curr. Med. Chem.* 25 984–1001. 28990516 10.2174/0929867324666171009121702

[B65] Ortega-Prieto P. Postic C. (2019). Carbohydrate sensing through the transcription factor ChREBP. *Front. Genet.* 10:472. 10.3389/fgene.2019.00472 31275349 PMC6593282

[B66] Palermo A. Maggi D. Maurizi A. R. Pozzilli P. Buzzetti R. (2014). Prevention of type 2 diabetes mellitus: is it feasible? *Diabetes Metab. Res. Rev.* 30(Suppl. 1), 4–12. 10.1002/dmrr.2513 24353270

[B67] Parzanese I. Qehajaj D. Patrinicola F. Aralica M. Chiriva-Internati M. Stifter S. (2017). Celiac disease: from pathophysiology to treatment. *World J. Gastrointest. Pathophysiol.* 8 27–38. 10.4291/wjgp.v8.i2.27 28573065 PMC5437500

[B68] Preidis G. A. Versalovic J. (2009). Targeting the human microbiome with antibiotics, probiotics, and prebiotics: gastroenterology enters the metagenomics era. *Gastroenterology* 136 2015–2031. 10.1053/j.gastro.2009.01.072 19462507 PMC4108289

[B69] Qin J. Li Y. Cai Z. Li S. Zhu J. Zhang F. (2012). A metagenome-wide association study of gut microbiota in type 2 diabetes. *Nature* 490 55–60. 23023125 10.1038/nature11450

[B70] Ramai D. Zakhia K. Fields P. J. Ofosu A. Patel G. Shahnazarian V. (2020). Fecal Microbiota Transplantation (FMT) with colonoscopy is superior to enema and nasogastric tube while comparable to capsule for the treatment of recurrent clostridioides difficile infection: a systematic review and meta-analysis. *Dig. Dis. Sci*. [Epub ahead of print] 10.1007/s10620-020-06185-7 32166622

[B71] Ridaura V. K. Faith J. J. Rey F. E. Cheng J. Duncan A. E. Kau A. L. (2013). Gut microbiota from twins discordant for obesity modulate metabolism in mice. *Science* 341:1241214. 10.1126/science.1241214 PMC3829625 24009397

[B72] Santos J. G. Alves B. C. Hammes T. O. Dall’alba V. (2019). Dietary interventions, intestinal microenvironment, and obesity: a systematic review. *Nutr. Rev.* 77 601–613. 10.1093/nutrit/nuz022 31188447

[B73] Scheithauer T. P. Dallinga-Thie G. M. De Vos W. M. Nieuwdorp M. Van Raalte D. H. (2016). Causality of small and large intestinal microbiota in weight regulation and insulin resistance. *Mol. Metab.* 5 759–770. 10.1016/j.molmet.2016.06.002 27617199 PMC5004227

[B74] Schwiertz A. Taras D. Schafer K. Beijer S. Bos N. A. Donus C. (2010). Microbiota and SCFA in lean and overweight healthy subjects. *Obesity* 18 190–195. 10.1038/oby.2009.167 19498350

[B75] Seddon P. J. Griffiths C. J. Soorae P. S. Armstrong D. P. (2014). Reversing defaunation: restoring species in a changing world. *Science* 345 406–412. 10.1126/science.1251818 25061203

[B76] Smits L. P. Bouter K. E. De Vos W. M. Borody T. J. Nieuwdorp M. (2013). Therapeutic potential of fecal microbiota transplantation. *Gastroenterology* 145 946–953. 10.1053/j.gastro.2013.08.058 24018052

[B77] Sonnenburg E. D. Zheng H. Joglekar P. Higginbottom S. K. Firbank S. J. Bolam D. N. (2010). Specificity of polysaccharide use in intestinal *Bacteroides* species determines diet-induced microbiota alterations. *Cell* 141 1241–1252. 10.1016/j.cell.2010.05.005 20603004 PMC2900928

[B78] Sonnenburg J. L. Sonnenburg E. D. (2019). Vulnerability of the industrialized microbiota. *Science* 366:eaaw9255. 10.1126/science.aaw9255 31649168

[B79] Soto M. Herzog C. Pacheco J. A. Fujisaka S. Bullock K. Clish C. B. (2018). Gut microbiota modulate neurobehavior through changes in brain insulin sensitivity and metabolism. *Mol. Psychiatry* 23 2287–2301. 10.1038/s41380-018-0086-5 29910467 PMC6294739

[B80] Swidsinski A. Loening-Baucke V. Lochs H. Hale L. P. (2005). Spatial organization of bacterial flora in normal and inflamed intestine: a fluorescence in situ hybridization study in mice. *World J. Gastroenterol.* 11 1131–1140. 10.3748/wjg.v11.i8.1131 15754393 PMC4250702

[B81] Tenorio-Jimenez C. Martinez-Ramirez M. J. Gil A. Gomez-Llorente C. (2020). Effects of probiotics on metabolic syndrome: a systematic review of randomized clinical trials. *Nutrients* 12:124. 10.3390/nu12010124 31906372 PMC7019472

[B82] Tilg H. Kaser A. (2011). Gut microbiome, obesity, and metabolic dysfunction. *J. Clin. Invest.* 121 2126–2132. 10.1172/jci58109 21633181 PMC3104783

[B83] Tilg H. Zmora N. Adolph T. E. Elinav E. (2020). The intestinal microbiota fuelling metabolic inflammation. *Nat. Rev. Immunol.* 20 40–54. 10.1038/s41577-019-0198-4 31388093

[B84] Turnbaugh P. J. Ley R. E. Mahowald M. A. Magrini V. Mardis E. R. Gordon J. I. (2006). An obesity-associated gut microbiome with increased capacity for energy harvest. *Nature* 444 1027–1031. 17183312 10.1038/nature05414

[B85] Ussar S. Griffin N. W. Bezy O. Fujisaka S. Vienberg S. Softic S. (2015). Interactions between gut microbiota, host genetics and diet modulate the predisposition to obesity and metabolic syndrome. *Cell Metab.* 22 516–530. 10.1016/j.cmet.2015.07.007 26299453 PMC4570502

[B86] Vallianou N. G. Stratigou T. Tsagarakis S. (2018). Microbiome and diabetes: where are we now? *Diabetes Res. Clin. Pract.* 146 111–118. 10.1016/j.diabres.2018.10.008 30342053

[B87] van Nood E. Vrieze A. Nieuwdorp M. Fuentes S. Zoetendal E. G. De Vos W. M. (2013). Duodenal infusion of donor feces for recurrent Clostridium difficile. *N. Engl. J. Med.* 368 407–415. 10.1056/nejmoa1205037 23323867

[B88] van Olden C. Groen A. K. Nieuwdorp M. (2015). Role of intestinal microbiome in lipid and glucose metabolism in diabetes mellitus. *Clin. Ther.* 37 1172–1177. 10.1016/j.clinthera.2015.03.008 25922340

[B89] Villanueva-Millan M. J. Perez-Matute P. Oteo J. A. (2015). Gut microbiota: a key player in health and disease. A review focused on obesity. *J. Physiol. Biochem.* 71 509–525. 10.1007/s13105-015-0390-3 25749935

[B90] Voshol P. J. Rensen P. C. Van Dijk K. W. Romijn J. A. Havekes L. M. (2009). Effect of plasma triglyceride metabolism on lipid storage in adipose tissue: studies using genetically engineered mouse models. *Biochim. Biophys. Acta* 1791 479–485. 10.1016/j.bbalip.2008.12.015 19168150

[B91] Vrieze A. Van Nood E. Holleman F. Salojarvi J. Kootte R. S. Bartelsman J. F. (2012). Transfer of intestinal microbiota from lean donors increases insulin sensitivity in individuals with metabolic syndrome. *Gastroenterology* 143:e917. 10.1053/j.gastro.2012.06.031 22728514

[B92] Wei Z. Shen P. Cheng P. Lu Y. Wang A. Sun Z. (2020). Gut bacteria selectively altered by sennoside A Alleviate Type 2 diabetes and obesity traits. *Oxid. Med. Cell Longev.* 2020:2375676. 10.1155/2020/2375676 PMC7334780 32685087

[B93] Weissman J. S. Coyle W. (2012). Stool transplants: ready for prime time? *Curr. Gastroenterol. Rep.* 14 313–316. 10.1007/s11894-012-0263-7 22585070

[B94] Wichmann A. Allahyar A. Greiner T. U. Plovier H. Lunden G. O. Larsson T. (2013). Microbial modulation of energy availability in the colon regulates intestinal transit. *Cell Host Microbe* 14 582–590. 10.1016/j.chom.2013.09.012 24237703

[B95] Woting A. Blaut M. (2016). The intestinal microbiota in metabolic disease. *Nutrients* 8: 202. 10.3390/nu8040202 27058556 PMC4848671

[B96] Wu G. D. Chen J. Hoffmann C. Bittinger K. Chen Y. Y. Keilbaugh S. A. (2011). Linking long-term dietary patterns with gut microbial enterotypes. *Science* 334 105–108. 10.1126/science.1208344 21885731 PMC3368382

[B97] Wu H. Ballantyne C. M. (2020). Metabolic inflammation and insulin resistance in obesity. *Circ. Res.* 126 1549–1564. 10.1161/circresaha.119.315896 32437299 PMC7250139

[B98] Xiao L. Chen B. Feng D. Yang T. Li T. Chen J. (2019). TLR4 may be involved in the regulation of colonic mucosal microbiota by Vitamin A. *Front. Microbiol.* 10:268. 10.3389/fmicb.2019.00268 30873131 PMC6401601

[B99] Yoon J. C. Chickering T. W. Rosen E. D. Dussault B. Qin Y. Soukas A. (2000). Peroxisome proliferator-activated receptor gamma target gene encoding a novel angiopoietin-related protein associated with adipose differentiation. *Mol. Cell Biol.* 20 5343–5349. 10.1128/mcb.20.14.5343-5349.2000 10866690 PMC85983

[B100] Yoshida N. Yamashita T. Hirata K. I. (2018). Gut microbiome and cardiovascular diseases. *Diseases* 6:56. 10.3390/diseases6030056 29966270 PMC6164700

[B101] Zhang F. Luo W. Shi Y. Fan Z. Ji G. (2012). Should we standardize the 1,700-year-old fecal microbiota transplantation? *Am. J. Gastroenterol.* 107 1755; author reply 1755–1756. 23160295 10.1038/ajg.2012.251

[B102] Zhang F. Wang M. Yang J. Xu Q. Liang C. Chen B. (2019). Response of gut microbiota in type 2 diabetes to hypoglycemic agents. *Endocrine* 66 485–493. 10.1007/s12020-019-02041-5 31410749

